# Exploring the potential use of melatonin as a modulator of tramadol-induced rewarding effects in rats

**DOI:** 10.3389/fphar.2024.1373746

**Published:** 2024-04-26

**Authors:** Alqassem Y. Hakami, Badrah S. Alghamdi, Fahad S. Alshehri

**Affiliations:** ^1^ College of Medicine, King Saud Bin Abdulaziz University for Health Sciences, Jeddah, Saudi Arabia; ^2^ King Abdullah International Medical Research Center, Jeddah, Saudi Arabia; ^3^ Department of Physiology, Neuroscience Unit, Faculty of Medicine, King Abdulaziz University, Jeddah, Saudi Arabia; ^4^ Neuroscience and Geroscience Research Unit, King Fahd Medical Research Center, King Abdulaziz University, Jeddah, Saudi Arabia; ^5^ Department of Pharmacology and Toxicology, College of Pharmacy, Umm Al-Qura University, Makkah, Saudi Arabia

**Keywords:** melatonin, tramadol, conditioned place preference, opioids, addiction

## Abstract

**Background:**

Melatonin is responsible for regulating the sleep-wake cycle and circadian rhythms in mammals. Tramadol, a synthetic opioid analgesic, is used to manage moderate to severe pain but has a high potential for abuse and dependence. Studies have shown that melatonin could be a potential modulator to reduce tramadol addiction.

**Methods:**

Male Wistar rats were used to investigate the effect of melatonin on tramadol-induced place preference. The rats were divided into four groups: control, tramadol, tramadol + melatonin (single dose), and tramadol + melatonin (repeated doses). Tramadol was administered intraperitoneally at 40 mg/kg, while melatonin was administered at 50 mg/kg for both the single dose and repeated-dose groups. The study consisted of two phases: habituation and acquisition.

**Results:**

Tramadol administration produced conditioned place preference (CPP) in rats, indicating rewarding effects. However, melatonin administration blocked tramadol-induced CPP. Surprisingly, repeated doses of melatonin were ineffective and did not reduce the expression of CPP compared to that of the single dose administration.

**Conclusion:**

The study suggests that melatonin may be a potential therapeutic option for treating tramadol addiction. The results indicate that melatonin attenuates the expression of tramadol-induced CPP, supporting its uses as an adjunct therapy for managing tramadol addiction. However, further studies are needed to investigate its effectiveness in humans.

## Introduction

The hormone melatonin, secreted by the pineal gland in the brain (Claustrat and Leston, 2015), plays a crucial role in regulating the sleep-wake cycle and circadian rhythms of mammals (Claustrat and Leston, 2015). Beyond these functions, melatonin exhibits a broad spectrum of physiological activities, including antioxidant activity, immunomodulation, and neuroprotection (Esposito and Cuzzocrea, 2010; Bantounou et al., 2022). Recent researches have explored its potential therapeutic benefits in sleep disorders, depression, anxiety, and drug addiction (Turek and Gillette, 2004; Papp et al., 2006; Cardinali et al., 2012; Onaolapo and Onaolapo, 2018; Alghamdi and Alshehri, 2021). Additionally, studies have investigated its efficacy in treating cardiovascular diseases, cancer, and Alzheimer’s disease (Sun et al., 2016; Li et al., 2017; Labban et al., 2021). Evidence also suggests that melatonin possesses anti-aging properties and can promote better sleep (Pereira et al., 2020; Bocheva et al., 2022).

Tramadol, a synthetic opioid analgesic, is widely prescribed for moderate to severe pain (Grond and Sablotzki, 2004; Subedi et al., 2019). Despite its effectiveness in pain management, tramadol carries a high potential for abuse and dependence (Roussin et al., 2015; Bassiony et al., 2017; Barbosa et al., 2023). The conditioned place preference (CPP) paradigm measures the drug-rewarding effects in animals (Huston et al., 2013). In CPP, animals learn to associate a specific location with the drug’s euphoric or dysphoric effects, and their preference for that location indicates the drug’s reinforcing properties (Bardo and Bevins, 2000). Studies have demonstrated that tramadol induces CPP in rodents, implying its rewarding effects (Epstein et al., 2006; Huston et al., 2013), which suggests a potential for misuse and abuse. Research has shown that tramadol use can lead to physical dependence and addiction, with individuals who have a history of substance abuse being particularly at risk of developing tramadol addiction (Epstein et al., 2006; Lanier et al., 2010).

The study of melatonin’s modulation of CPP in response to tramadol in rats is critically important, given the escalating concerns about tramadol misuse and abuse (Wood and Dargan, 2021). Tramadol, a commonly prescribed opioid analgesic, is linked to addiction, dependence, and various adverse effects (Reines et al., 2020). Consequently, it is vital to investigate drugs that reduce tramadol’s rewarding effects to prevent addiction and its associated issues. Previous research indicates that melatonin might be an effective treatment for diminishing the effects of several drug addictions (Hemati et al., 2021). Recently, it has been shown that melatonin can block morphine induced CPP through modulating glutamate transporter −1 (GLT-1) and brain-derived neurotrophic factor (BDNF), nuclear factor-kappa B (NF-κB), and cAMP response element-binding protein CREB expression levels (Alghamdi and Alshehri, 2021; Alshehri et al., 2021). Previous research showed evidence that alcohol-dependent humans and rodents experience reduction in melatonin levels and delay in reaching their nocturnal peak concentration of melatonin and activating melatonin receptors using melatonin or agomelatine reduced alcohol seeking in rats (Vengeliene et al., 2015). Other studies have shown melatonin can reduce cocaine (Takahashi et al., 2017), methamphetamine (Clough et al., 2016), and fentanyl seeking behavior (Du et al., 2024). Thus, exploring melatonin’s impact on tramadol-induced CPP in rats is imperative to assess its therapeutic potential for tramadol addiction management.

Melatonin has demonstrated potential effects against the rewarding properties of various drugs of abuse, such as cocaine and morphine, in animal models (Takahashi et al., 2017). However, the impact of melatonin on tramadol-induced CPP remains underexplored. Therefore, this study investigated melatonin as a potential therapeutic compound for treating tramadol addiction.

## Materials and methods

### Animals

Male Wistar rats weighing 250–300 g were utilized. They were housed in pairs under a 12:12 light/dark cycle, with *ad libitum* access to food and water. All animals were handled by expert researchers and housed in pairs to minimize stress. The study received approval from the King Fahd Medical Research Center Animal Care and Use Committee. Furthermore, the Biomedical Ethics Research Committee at the King Abdulaziz University (Reference 405-20) the experiments in accordance with the ethical guidelines and research protocols for living organisms established by the King Abdulaziz City for Science and Technology, as authorized by the Royal Decree No. M/59 on 24 August 2010.

### Drugs

Tramadol hydrochloride (Sigma Aldrich, USA) and melatonin (Sigma Aldrich, USA), were freshly prepared daily, using 0.5% ethanol and diluted with saline to serve as the vehicle (i.p. 1 mL/kg).

### Experimental design and dosing

#### Phase I

**FIGURE 1:**
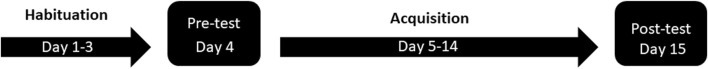
Timeline of the CPP experiment showing the habituation, pre-test, acquisition and post-test.

Figure 1 illustrates the habituation phase spanning from day 1 to day 3. Throughout this phase, the animals were allowed to explore the open apparatus for a total of 20 min each day. On day 4, a 20-min pre-test was performed to assess the animals’ preference. The OPTO-MAX Auto-Track software documented various parameters, including the duration, overall activity count, ambulatory count, rest time, and distance covered by the animals in each chamber. A preference for the black chamber was observed among the majority of animals, necessitating the use of a biased approach.

#### Phase II

During the acquisition phase, from day 5 to day 14, each animal was placed in the assigned chamber for 45 min. The post-test was conducted on day 15 for 20 min. During this test, the apparatus was open to the animals for a total of 20 min, and the CPP score was calculated. Animals were euthanized using isoflurane on day 16.

### Animal groups and dosing

Four groups of animals, each comprising approximately 6-8 rats as detailed in Table 1, were divided as follows: (1) control, (2) tramadol, (3) tramadol + melatonin (single dose), and (4) tramadol + melatonin (repeated doses). The control group was administered vehicle injections throughout the experiment. The tramadol group received tramadol injections (40 mg/kg, i.p.) on alternate days, totaling five injections. The tramadol + melatonin (single dose) group was administered tramadol injections (40 mg/kg, i.p.) on alternate days for five injections, with a single dose of melatonin (50 mg/kg, i. p.) administered 30 min before the post-test. The tramadol + melatonin (repeated doses) group received concurrent injections of tramadol (40 mg/kg, i.p.) and melatonin (50 mg/kg, i.p.) on alternate days, also totaling five injections.

**TABLE 1:** Animals groups and treatment.

Groups	Treatment
Control	Vehicle
Tramadol	Tramadol (40 mg/kg, i.p)
Tramadol + melatonin (single dose)	Tramadol (40 mg/kg, i.p)+Melatonin (50 mg/kg, i.p) single dose before post-test
Tramadol + melatonin (Repeated doses)	Tramadol (40 mg/kg, i.p)+Melatonin (50 mg/kg, i.p), five doses during acquisition

### CPP score, total activity, ambulatory count, resting time, and distance traveled

The test utilized a three-compartment apparatus constructed from Plexiglas (Columbus Instruments in Columbus, OH, USA). It comprised two main chambers, each featuring unique visual cues and flooring textures. The white chamber was marked by vertical white stripes and a smooth white floor, whereas the black chamber displayed a pattern of small white and black squares and had a small circle drilled into the floor. A smaller external chamber situated between these two served as a separator. Infrared sensors tracked the animals’ movements and activity throughout the CPP test. The test was recorded for 20 min and all parameters were recorded including the time spent in each chamber, total activity, ambulatory count, resting time, and distance traveled.

### Data analysis

All data comprising the CPP score, such as the distance traveled, resting time, ambulatory count, and total activity count, were analyzed using a repeated measure ANOVA followed by the Tukey’s *post hoc* test. Statistical significance was set at *p* < 0.05. GraphPad Prism version 10.2.1 was used to analyze the results and create the figures.

## Results

**FIGURE 2:**
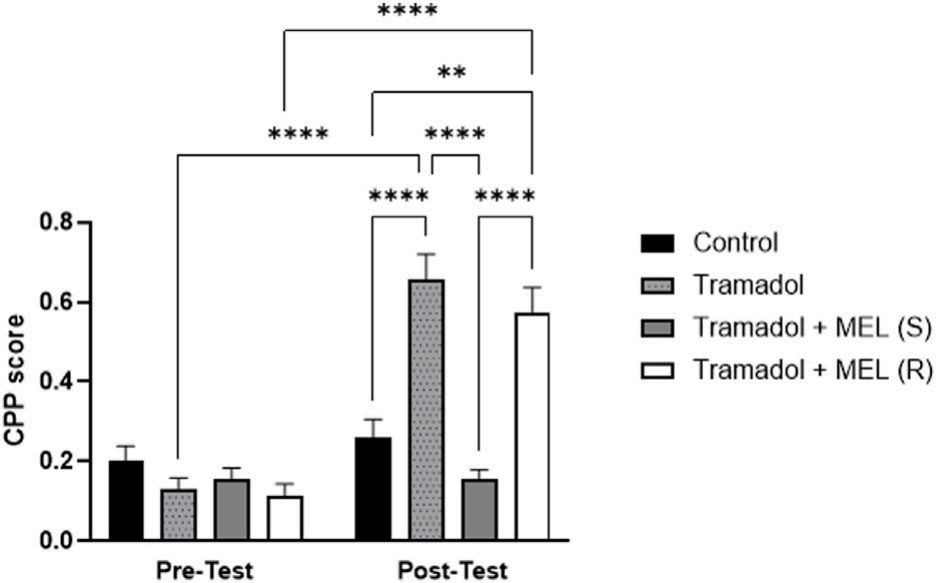
CPP score for the effect of melatonin and tramadol in CPP on all groups (control, tramadol, tramadol + melatonin “single dose”, and tramadol + melatonin “repeated doses”).

The primary objective of this study was to investigate the effect of melatonin on tramadol-induced CPP. We aimed to determine whether melatonin administration could attenuate the CPP induced by tramadol in rats Figure 2. Repeated measures ANOVA revealed significant effect of the days (F (1, 7) = 78.4, *p* < 0.0001), the effect of treatment (F (3, 21) = 22.79, *p* < 0.0001), and the interaction between treatment and days (F (3, 21) = 17.82, *p* < 0.0001). Further analysis using Tukey’s *post hoc* test indicated a significant increase in CPP scores for animals that received five doses of tramadol during the acquisition phase (tramadol group) compared to the control group (*p* < 0.0001). However, administering melatonin 30 min before the post-test prevented the tramadol-induced CPP (*p* < 0.0001). In contrast, repeated doses of melatonin given with tramadol during the acquisition phase did not reduce tramadol-seeking behavior during the post-test (*p* = 0.5188). Also, *post hoc* test showed significant increase in CPP score comparing pre-test and post-test in tramadol group (*p* < 0.0001) and pre-test and post-test in tramadol + MEL (R) group (*p* < 0.0001).

**FIGURE 3:**
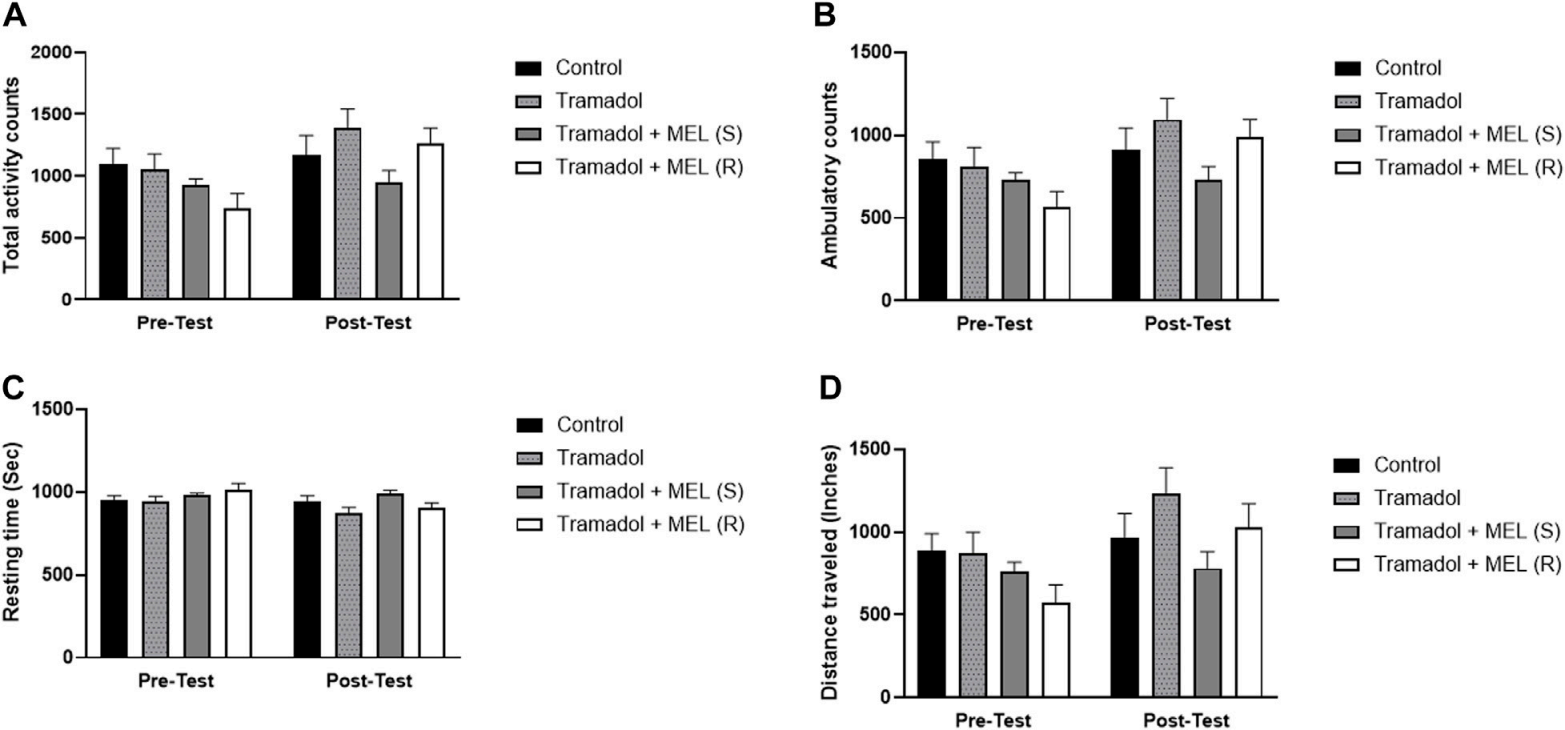
**(A)** Total activity, **(B)** ambulatory count, **(C)** resting time, and **(D)** distance traveled for the effect of melatonin and tramadol in CPP on all groups (control, tramadol, tramadol + melatonin “single dose”, and tramadol + melatonin “repeated doses”).

In addition to evaluating melatonin’s impact on tramadol-induced CPP, we performed further analyses to examine additional relevant parameters that might influence our results, Figure 3. We quantified the total activity, ambulatory count, resting time, and distance traveled. These measures enabled more comprehensive understanding of the animal’s behavior and more precise assessment of melatonin’s effects.

First, we assessed melatonin’s impact on tramadol’s total activity, Figure 3A. Repeated measures ANOVA showed significant effect on days (F (1, 7) = 9.321, *p* = 0.0185), no treatment effect (F (3, 21) = 2.138, *p* = 0.1258), and no interaction between treatment and days (F (3, 21) = 2.259, *p* = 0.1113). Second, we evaluated melatonin’s influence on tramadol’s ambulatory count, Figure 3B. Repeated measures ANOVA indicated significant effect on days (F (1, 7) = 8.343, *p* = 0.0234), no treatment effect (F (3, 21) = 2.033, *p* = 0.1400), and no effect of treatment and days (F (3, 21) = 2.422, *p* = 0.0944).

Third, we assessed the impact of melatonin on tramadol-induced resting time, Figure 3C. Repeated measures ANOVA indicated significant effects over days (F (1, 7) = 6.787, *p* = 0.0352), no significant treatment effect (F (3, 21) = 2.268, *p* = 0.1103), and no interaction between treatment and days (F (3, 21) = 2.199, *p* = 0.1182). Lastly, we evaluated the influence of melatonin on the distance traveled, Figure 3D. Repeated measures ANOVA showed significant effects on days (F (1, 7) = 9.295, *p* = 0.0186), no significant treatment effect (F (3, 21) = 2.261, *p* = 0.1110), and no interaction between treatment and days (F (3, 21) = 2.250, *p* = 0.1123).

## Discussion

Several preclinical studies have reported the rewarding outcomes of opioids using CPP and other behavioral techniques, such as self-administration (Sim-Selley et al., 2000; Zhang et al., 2012; Mavrikaki et al., 2017; Reeves et al., 2021). Moreover, CPP is considered as one of the most popular non-invasive models for measuring the motivational effects of drugs of abuse in experimental animals (Mavrikaki et al., 2017). Previous reports have consistently demonstrated the rewarding effects of tramadol using the CPP technique (Abdel-Ghany et al., 2015; Sadeghi-Adl et al., 2020; Barbosa et al., 2023). Melatonin, a hormone synthesized by the pineal gland, is essential for maintaining the regular circadian rhythm in mammals (Dubocovich, 2007). Notably, melatonin has played a potential role in attenuating the seeking behavior for several drugs of abuse (Kovanen et al., 2010; Conroy et al., 2012; Alghamdi and Alshehri, 2021; Alshehri et al., 2021). The CPP paradigm can provide further insights into animal behavior beyond the time spent in each chamber, such as the resting time, total activity, ambulatory count, and total distance.

Tramadol, an opioid analgesic, has shown potential for abuse according to epidemiological evidence, coinciding with the increased global demand for opioids over the past 2 decades (Berterame et al., 2016; Dunn et al., 2019). Tramadol also induces physical dependence and withdrawal syndrome upon discontinuation, similar to other opioids (Carroll et al., 2006; Lofwall et al., 2007). Specifically, it has been demonstrated to produce a CPP rewarding effect in rats (Sprague et al., 2002; Tzschentke et al., 2002). Moreover, tramadol affects multiple neurotransmitter systems, including serotonin and norepinephrine, and its effects are partially antagonized by naloxone (Desmeules et al., 1996; Apaydin et al., 2000). Notably, an *in vivo* microdialysis study provided evidence of a statistically significant increase in dopamine release within the nucleus accumbens shell following a tramadol suggesting preclinical evidence of tramadol’s rewarding effects within the reward circuit (Asari et al., 2018). Consistent with previous findings, this study demonstrated the CPP rewarding effects with tramadol administration in rats.

Studies on melatonin have demonstrated a decrease in dopamine release, primarily through effects on dopamine receptors (Zisapel et al., 1982; Zisapel, 2001). Furthermore, stimulation of melatonin receptors has been shown to reduce alcohol relapse-like behavior in Wistar rats (Vengeliene et al., 2015). A single dose of melatonin significantly attenuated the expression of tramadol-induced CPP. This finding aligns with those of a previous report from our laboratory, which found that administering melatonin 30 min before morphine treatment diminished the morphine CPP effect (Alghamdi and Alshehri, 2021). The same study also revealed that melatonin reversed the expression levels of GLT-1, NF-κB, CREB, and BDNF. Similarly, other studies have indicated that melatonin can restore neuronal impairment induced by methamphetamine in mice (Veschsanit et al., 2021). Therefore, melatonin is recognized for attenuating the rewarding effects and modulating the neuronal impairment caused by drugs of abuse.

Conducting the CPP test during the day, rather than at night, aligns with the pharmacological properties and mechanisms of action of melatonin. Melatonin is a hormone primarily secreted by the pineal gland in response to darkness, with levels typically peaking at night to regulate the sleep-wake cycle and synchronize circadian rhythms in Wistar rats (Sánchez et al., 2004; Sánchez et al., 2008). By administering melatonin and conducting the CPP test during the day is to minimize any potential confounders associated with melatonin release during the night and ensuring the reliability of the results. Furthermore, this study performed several tests including (total activity, ambulatory count, resting time, and distance traveled) to assure the melatonin doses during the day does not affect CPP tests.

The study’s observation that repeated doses of melatonin did not reduce tramadol-seeking behavior during the post-test underscores the complexity of the interaction between melatonin and tramadol in the context of CPP. The initial administration of melatonin 30 min before the acquisition phase test successfully attenuated the seeking behavior induced by tramadol. However, the efficacy of melatonin appeared to diminish with repeated administration. Tramadol is typically considered a mild μ-receptor agonist and also affects other neurotransmitter systems, including serotonergic, noradrenergic, and gamma-aminobutyric acid systems (Bamigbade et al., 1997; Gillen et al., 2000; Jesse et al., 2010). The mechanisms by which tramadol influences each of these systems remain unclear, and limited research is available.

Melatonin produces analgesic properties through a variety of biological pathways (Ambriz-Tututi et al., 2009). Animals and humans studies have demonstrated its efficacy in alleviating nociceptive and neuropathic pain (Srinivasan et al., 2012; Borsani et al., 2017; Kuthati et al., 2019; Shokri et al., 2021). In rodent models, melatonin shows antinociceptive and anti-hyperalgesic effects against a range of stimuli, including inflammation and nerve injury (Yu et al., 2000; Ulugol et al., 2006; Posa et al., 2018). These effects are believed to be a result of the activation of melatonin receptors present in critical pain-regulating regions such as the spinal cord, thalamus, and hypothalamus (Laurido et al., 2002; Lopez-Canul et al., 2015). Activation of these receptors results in the reduction of cyclic AMP levels and inhibition of Ca2+ channels (Vanecek and Vollrath, 1989), consequently lowering intracellular Ca2+ levels (Vanecek, 1995; Vanecek and Watanabe, 1998), which are essential in the central sensitization process associated with inflammatory and neuropathic pain. Furthermore, melatonin modulates various receptor systems, including dopaminergic (Abilio et al., 2003), GABAergic (Golombek et al., 1996), opioidergic (Hemati et al., 2021), and serotonergic pathways (Valdes-Tovar et al., 2018). Melatonin shows anti-inflammatory and antioxidative characteristics (Nabavi et al., 2019; Bantounou et al., 2022), further enhancing its analgesic efficacy (Burchakov and Uspenskaya, 2019). Acting as a potent free radical scavenger, melatonin neutralizes reactive oxygen and nitrogen species and facilitate the activity of antioxidative enzymes such as glutathione peroxidase and superoxide dismutase (Tsia and Hu, 2003; Reiter et al., 2007). On the other hand, tramadol use have been reported to be associated with the activation of proinflammatory cytokines (Kraychete et al., 2009), and glutamatergic involvement (Chetan et al., 2015). Thus, the complex mechanisms of melatonin together with tramadol reduce seeking behavior associated with tramadol use. Using melatonin to attenuate the seeking behavior of tramadol offers advantages in regulating sleep patterns disrupted by tramadol use (Abdullah et al., 2020), and neuroprotective properties, though its direct efficacy in countering tramadol induce CPP. On the other hand, naloxone, as an opioid receptor antagonist, directly blocks opioid effects of tramadol, which may help prevent Tramadol induction of CPP; however, naloxone will participate in the withdrawal symptoms in physically dependent individuals which could limit its suitability for tramadol since tramadol is weak mu opioids against (Lagard et al., 2018). Lastly, the choice between melatonin and naloxone depends on factors such as the severity of addiction, comorbid conditions, and treatment goals.

In this study, various behavioral tests such as total activity, ambulatory count, resting time, and distance traveled serve as important measurement for assessing the rewarding or aversive properties of environmental stimuli. Total activity provides a comprehensive measure of overall locomotor behavior, reflecting the general stimulation level of the rats. Ambulatory count specifically quantifies voluntary movements, to understand the exploratory behavior and activity patterns within the test environment. Resting time, conversely, shows periods of inactivity or grooming potentially indicating the presence of preferred or aversive behavior. Distance traveled serves as a cumulative measure of the spatial exploration undertaken by the subject throughout the conditioning process. These tests collectively contribute to explaining the subtle behavioral responses associated with conditioned preferences, offering valuable insights into the underlying mechanisms of reward and aversion processing.

Limitation, the findings highlight the need to determine the optimal dosing regimen for melatonin and to ascertain whether its impact on drug-seeking behavior diminishes over time, necessitating further research into the dynamics of this interaction. Understanding how melatonin influences tramadol in a repeated dosing context is crucial for explaining its potential therapeutic uses. Additionally, investigating the molecular and neurobiological alterations that occur with chronic melatonin administration in conjunction with tramadol may reveal mechanisms underlying the observed effects. In summary, the immediate influence of melatonin on tramadol-induced CPP underscores the importance of thoroughly understanding the temporal aspects and dose-response relationships to fully understand melatonin’s potential to attenuate tramadol’s drug-seeking behavior

## Conclusion

The results of this study suggest that melatonin may offer therapeutic benefits in treating tramadol addiction. Administration of melatonin significantly reduced the expression of tramadol-induced CPP in rats. Furthermore, analysis of relevant parameters, including the total activity, ambulatory count, resting time, and distance traveled, revealed that melatonin did not significantly affect these measures. Thus, the influence of melatonin on tramadol-seeking behavior appears to be specific and not attributable to changes in the overall activity or locomotion. These findings support the potential use of melatonin as an adjunct therapy for managing tramadol addiction, although further research is necessary to assess its efficacy in humans. This study highlights the importance of investigating potential pharmacological interventions for drug addiction treatment and offers valuable insights into the neurobiological mechanisms of tramadol addiction.

## Data Availability

The raw data supporting the conclusion of this article will be made available by the authors, without undue reservation.
